# Nosema bombycis microRNA-like RNA 8 (Nb-milR8) Increases Fungal Pathogenicity by Modulating *BmPEX16* Gene Expression in Its Host, Bombyx mori

**DOI:** 10.1128/Spectrum.01048-21

**Published:** 2021-10-27

**Authors:** Zhanqi Dong, Ning Zheng, Congwu Hu, Boyuan Deng, Wenxuan Fang, Qin Wu, Peng Chen, Xuhua Huang, Na Gao, Cheng Lu, Minhui Pan

**Affiliations:** a State Key Laboratory of Silkworm Genome Biology, Southwest University, Chongqing, China; b Key Laboratory of Sericultural Biology and Genetic Breeding, Ministry of Agriculture and Rural Affairs, Southwest University, Chongqing, China; c The General Extension Station of Sericulture Technology of Guangxi Zhuang Autonomous Region, Nanning, China; Broad Institute

**Keywords:** *BmPEX16*, *Bombyx mori*, *Nb-milR8*, *Nosema bombycis*, milRNAs

## Abstract

The fungus Nosema bombycis causes significant economic losses via parasitism of an economically important insect. MicroRNAs (miRNAs) play important roles in regulating host and parasite gene expression via mRNA degradation or by inhibiting protein translation. To investigate whether microRNA-like RNAs (milRNAs) regulate *N. bombycis* pathogenesis and to better understand the regulatory mechanisms underlying infection, we constructed small RNA libraries from *N. bombycis* hyphae during the schizont proliferation period. Eleven novel milRNAs were determined by RNA sequencing and stem-loop reverse transcriptase PCR (RT-PCR) assays. Moreover, a virulence-associated milRNA, Nb-milR8, was identified as critical for *N. bombycis* proliferation by binding and downregulating expression of its target gene, *BmPEX16*, in the host during infection. Silencing of Nb-milR8 or overexpression of the target *BmPEX16* gene resulted in increased susceptibility of Bombyx mori to *N. bombycis* infection. Taken together, these results suggest that Nb-milR8 is an important virulence factor that acts as an effector to suppress host peroxidase metabolism, thereby facilitating *N. bombycis* proliferation. These results provide important novel insights into interactions between pathogenic fungi and their hosts.

**IMPORTANCE** A thorough understanding of fungal pathogen adaptations is essential for treating fungal infections. Recent studies have suggested that the role of small RNAs expressed in fungal microsporidia genomes are important for elucidating the mechanisms of fungal infections. Here, we report 11 novel microRNA-like RNAs (milRNAs) from the fungal microsporidium Nosema bombycis and identified NB-milRNAs that adaptively regulate *N. bombycis* proliferation. In addition, we demonstrate that *N. bombycis* modulates small RNA (sRNA)-mediated infection by encoding an Nb-miR8 that downregulates the expression of the host peroxidase metabolism protein BmPEX16, which is essential for peroxisome membrane biogenesis and peroxisome assembly. These results significantly contribute to our understanding of the pathogenic mechanisms of fungi, and especially microsporidia, while providing important targets for genetical engineering-based treatment of microsporidia.

## INTRODUCTION

Microsporidia are obligate intracellular parasitic unicellular eukaryotes (1–3). Among 200 microsporidia genera, 93 have insect hosts and are important biological control agents of agricultural pests and important pathogens of economically relevant insects (2, 4). Most parasitic pathogens have sublethal effects on their insect hosts and contribute to reduced fertility, shortened life spans, and loss of vitality (4, 5). Therefore, understanding the physiological characteristics of microsporidia and the relationship between microsporidia and their hosts are important for understanding the roles of microsporidia in agricultural production. Sequencing of microsporidia genomes has led to several investigations that have primarily focused on evaluating genomic sequence, gene evolution, and molecular function, thereby providing useful baseline data and knowledge for preventing microsporidia infections and understanding the mechanistic basis of their investigations (6, 7). Nevertheless, manipulation of microsporidia genomes by genetic modification has been minimally studied, and comprehensive treatment of microsporidia has remained elusive.

Increasing attention has been paid in recent years to understanding microRNAs (miRNAs) that are transcribed, but do not encode proteins, and can perform biological functions at the RNA level within pathogen-host interactions (8–10). miRNAs are short RNA sequences with lengths typically of 18 to 25 nucleotides (nt) and are endogenous noncoding RNAs with regulatory functions that primarily exist in eukaryotes (11). Lee et al. identified an miRNA-like RNA (milRNA) in Neurospora crassa in 2010 with similar characteristics to animal and plant milRNAs and observed that milRNA production was dependent on *Dicer* enzyme cleavage in addition to the activities of Dicer-like 1 (DCL-1) and other key enzymes (12). milRNAs are primarily involved in fungal stress responses, morphological regulation, and a variety of physiological processes (13–15). Most milRNAs can induce gene silencing but also inhibit host immune responses (16). For example, Fusarium oxysporum *f.* sp. *lycopersici* (Fol) Fol-milR1 is a fungal effector that suppresses host immunity by silencing a disease resistance gene in tomato plants (17). Further, some miRNAs act as virulence factors to regulate host or pathogen factors, including the fungus Valsa mali Vm-milRNA-16, which contributes to *V. mali* infection of apple trees by adaptively regulating virulence genes, and VA-milRNAs are virulence factors associated with infection of Rhizoctonia solani direct interference with host target genes (18, 19).

Nosema bombycis is the first described microsporidium of insect pathogens (20). The pathogen has caused huge economic losses in the Chinese and European sericulture industry, leading to the near-disappearance of European sericulture (20). Indeed, it is the primary pathogen within the sericulture industry of China, India, and Southeast Asia. *N. bombycis* exhibits unique biological characteristics for a eukaryote, including ribosomal similarities to those of prokaryotes (21). Given that miRNA widely exists in eukaryotic cells, it is important to explore whether *N. bombycis* milRNA is important for modulating its biological characteristics. Importantly, the functional and mechanistic characteristics of *N. bombycis* pathogenic action have not been elucidated, and it is thus a considerable challenge to identify the molecular mechanisms of interactions between these fungi and their hosts.

In this study, we identified milRNA in *N. bombycis* to help identify the pathogenic mechanism of *N. bombycis*, while further analyzing the mechanism of milRNAs and their target genes that regulate the proliferation of *N. bombycis*. We first analyzed the infective characteristics of *Bombyx mori* embryonic cell line BmE-SWU1 infected with *N. bombycis* by transmission electron microscopy (TEM), immunofluorescence, and reverse transcriptase PCR (RT-PCR). High-throughput whole-transcriptome sequencing was then used to identify RNA profiles of infection during schizont proliferation and abundant spore replication phases at 48 h postinfection (h p.i.). A total of 11 novel milRNAs in *N. bombycis* were identified, including Nb-milR1 to Nb-milR12 (excluding Nb-milR7) using high-throughput sequencing and stem-loop RT-PCR analysis. Moreover, functional characterization of Nb-milR8 and its corresponding target gene, Bombyx mori peroxisomal membrane protein (*BmPEX16*), indicates that milRNAs can adaptively regulate host genes to promote *N. bombycis* proliferation. These results provide evidence for the existence of milRNAs in *N. bombycis* that may provide novel insights into milRNAs, in addition to the molecular mechanisms of fungal pathogen-host interactions.

## RESULTS

### Proliferation of *N. bombycis*.

**FIG 1:**
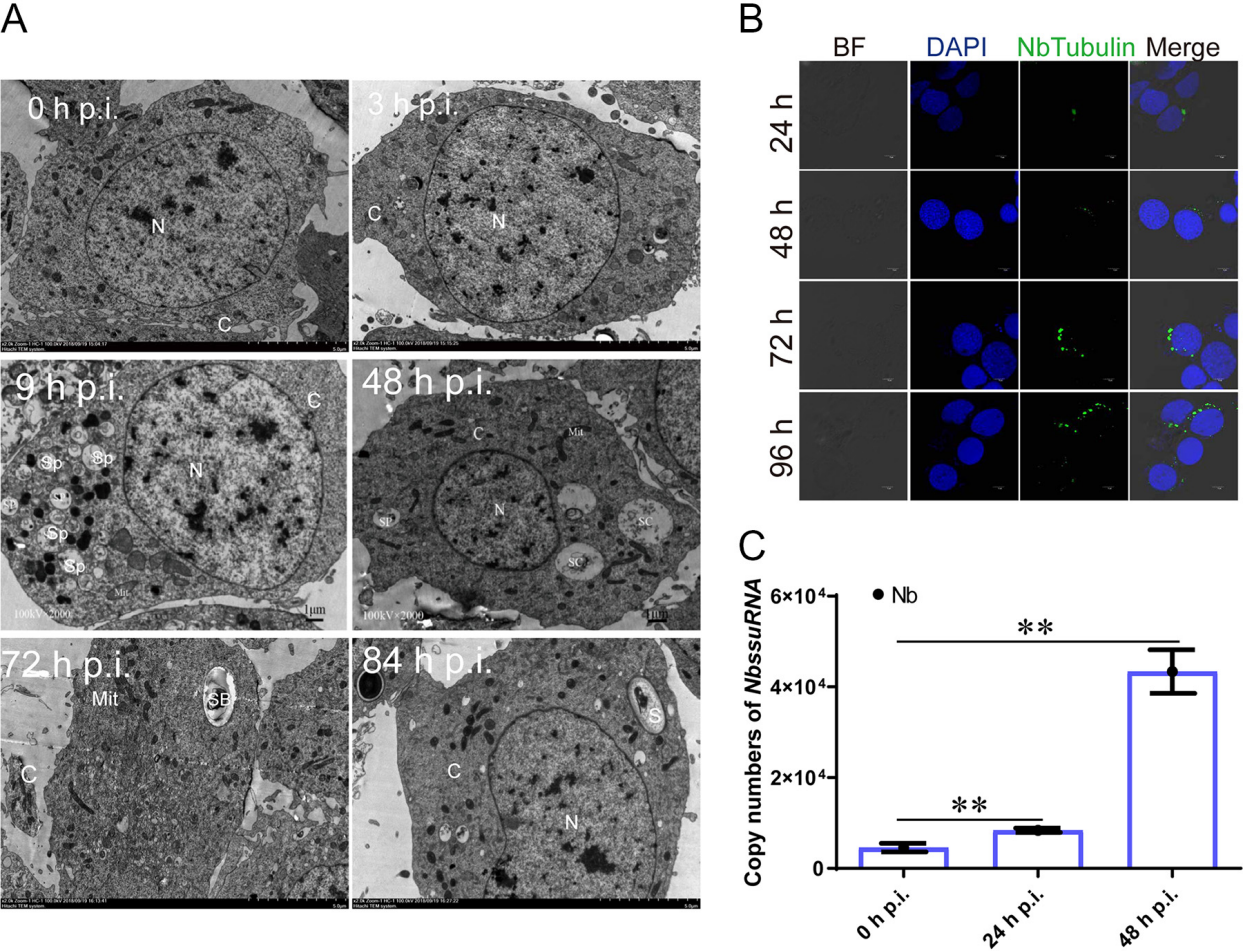
Infection proliferation characteristics of *N. bombycis.* (A) TEM micrographs illustrating the *N. bombycis* life cycle in BmE-SWU1 cells. N, nucleus; C, cytoplasm; Sp, spore plasm; Sc, schizont; Sb, sporoblast; S, spore. (B) Immunofluorescence assays showing *N. bombycis* proliferation characteristics. BF, bright fluorescence; DAPI shows *B. mori* and *N. bombycis* nuclei; green fluorescence shows the NbTubulin proteins. (C) RT-PCR analysis of *N. bombycis* genomic DNA copies in BmE-SWU1 cells, based on Nb SSURNA copy numbers. All data represent means of three replicates ± SD. **, *P < *0.01.

To better predict and analyze milRNA characteristics of *N. bombycis*, the life cycle of *N. bombycis* was first explored with immunofluorescence, gene expressional analysis, and TEM analysis. TEM visualization indicated that spore plasms could be observed at 3 h p.i., while spore plasm volumes increased at 9 h p.i., and bi-nucleated schizonts appeared at 48 h p.i. (Fig. 1A). The changes were followed by sporocyst appearances, cell membrane thickening, and chitinous wall formation at 72 h p.i. (Fig. 1A). Finally, mature microspores with complete walls and polar tube structures appeared at 84 h p.i. (Fig. 1A). Immunofluorescence was performed by labeling Nb-tubulin proteins, revealing that a small number of spores were injected into the cytoplasm at 24 h p.i. by ejecting polar filaments and a small amount of spore protoplasm (Fig. 1B). Subsequently, the spore plasms became larger and developed into schizonts through two divisions at 48 h p.i. RT-PCR was used to analyze genome copy numbers after *N. bombycis* infection. Genomic DNA copies significantly increased in number at 48 h p.i. (Fig. 1C). Thus, *N. bombycis* was in the schizont stage at 48 h p.i., based on the presence of a larger number of *N. bombycis* genome copies due to replication. This period was thus identified as a key time for screening miRNA.

### Identification of milRNAs in *N. bombycis*.

**FIG 2:**
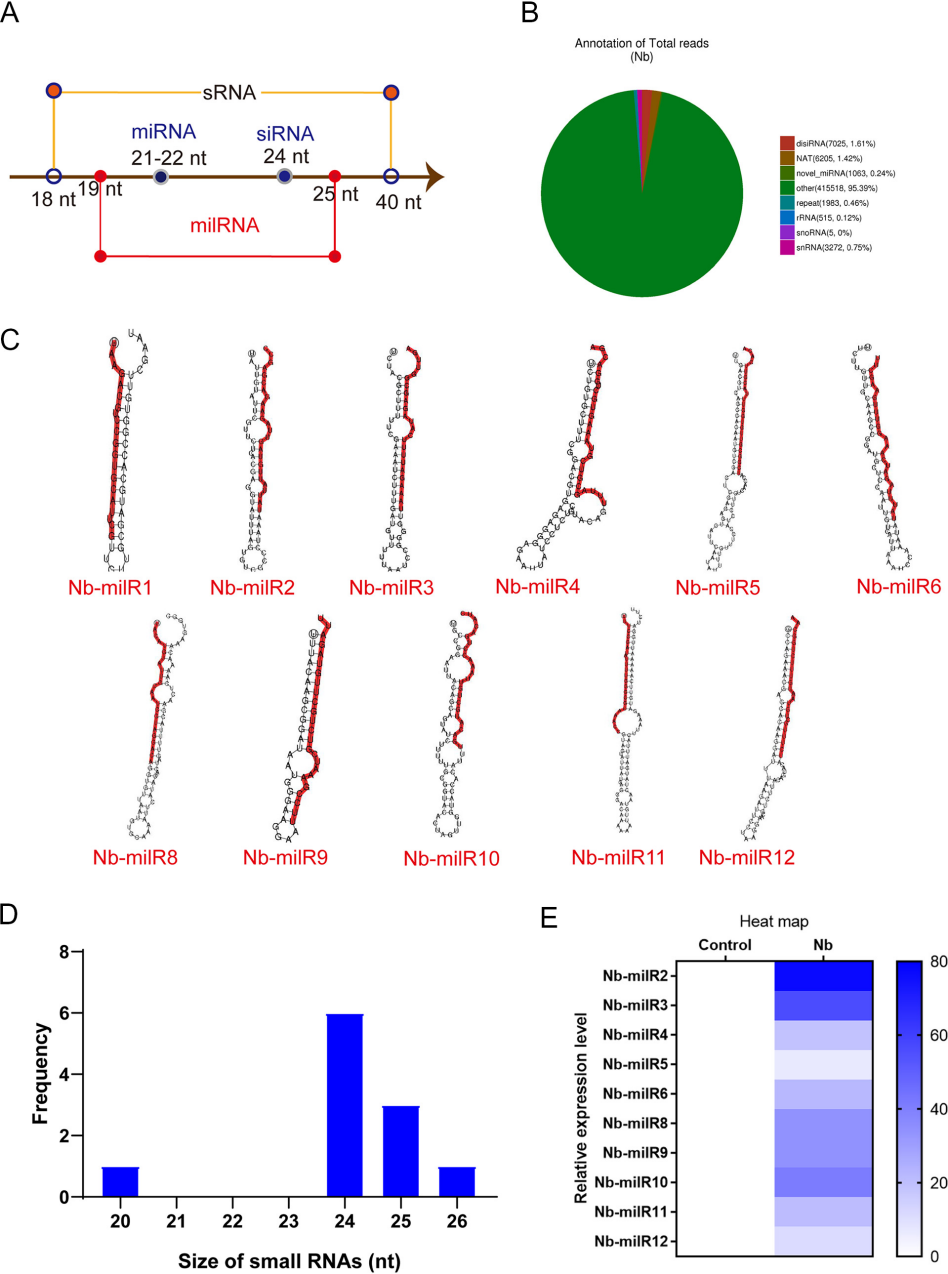
Identification of MicroRNA-like RNAs (milRNA) in *N. bombycis*. (A) The length distributions of *N. bombycis* sRNAs. (B) The relative abundances of different sRNA classes in *N. bombycis*. (C) Hairpin structure characteristics for the Nb-miRNA precursor. Red lines indicate the sequences of mature Nb-milRNAs. (D) The length distribution for Nb-milRNAs in *N. bombycis*. (E) Expression levels of different Nb-milRNAs in *N. Bombycis*. All data represent the means of three replicates ± SD. **, *P < *0.01.

To predict *N. bombycis* milRNAs in BmN-SWU1 cells, RNA from the *N. bombycis* infected at 48 h p.i. (Nb) and uninfected (control) groups were subjected to Illumina Solexa high-throughput sequencing. A total of 12,657,370 and 19,108,751 reads were generated in the Nb and control groups, respectively, with more than 95% of reads exhibiting greater than Q20 or Q30 qualities. Thus, the sequencing data were of high quality and could be used for further analyses. Low-quality sequences, 5′ terminal junction contaminated sequences, and sequences without 3′ terminal junctions and insertion fragments were all quality filtered and removed to generate the final sequence data sets. After quality filtering, a total of 12,081,520 and 18,766,136 sRNA clean reads were obtained for the Nb and control groups, respectively (see Table S2 in the supplemental material). Clean reads for each sample were used to identify sRNAs within certain length ranges for subsequent analyses. sRNAs primarily exhibit 18- to 40-nucleotide (nt) lengths, while miRNA is 21 to 22 nt long, and milRNAs are longer than miRNAs (Fig. 2A and Fig. S1). Using this framework, milRNAs were identified based on lengths of clean reads that were obtained from the Nb group. milRNAs accounted for 0.24% of the samples when considering all sRNAs and all RNA data sets (Fig. 2B).

The signature hairpin structures of miRNA precursors can be used to predict novel miRNAs. Thus, the miREvo and mirdeep2 miRNA prediction software programs were used to identify novel miRNAs (22, 23). The basic principle of miRNA identification is to probe certain lengths of sRNA alignment reference sequences, followed by analysis of secondary structures, dicer site information, free energy, and other characteristics. Subsequently, the sequence, compositions, lengths, and frequencies of sRNAs in each sample were analyzed. Among the potential Nb-milRNA genes of *N. bombycis* that were predicted, 11 novel miRNAs were identified, and their secondary structures were analyzed (Fig. 2C). The size distributions of the different Nb-milRNAs were also analyzed. Nb-milRNAs primarily exhibited lengths of 20 to 26 nt in *N. bombycis*. Over 80% of the milRNAs exhibited lengths of 24 to 25 nt (Fig. 2D). The 10 novel Nb-milRNAs were only mapped to *N. bombycis* (Fig. 2E and Fig. S1B), while Nb-milR1 was mapped to both groups.

### Transcriptional expression of *N. bombycis* milRNAs.

**FIG 3:**
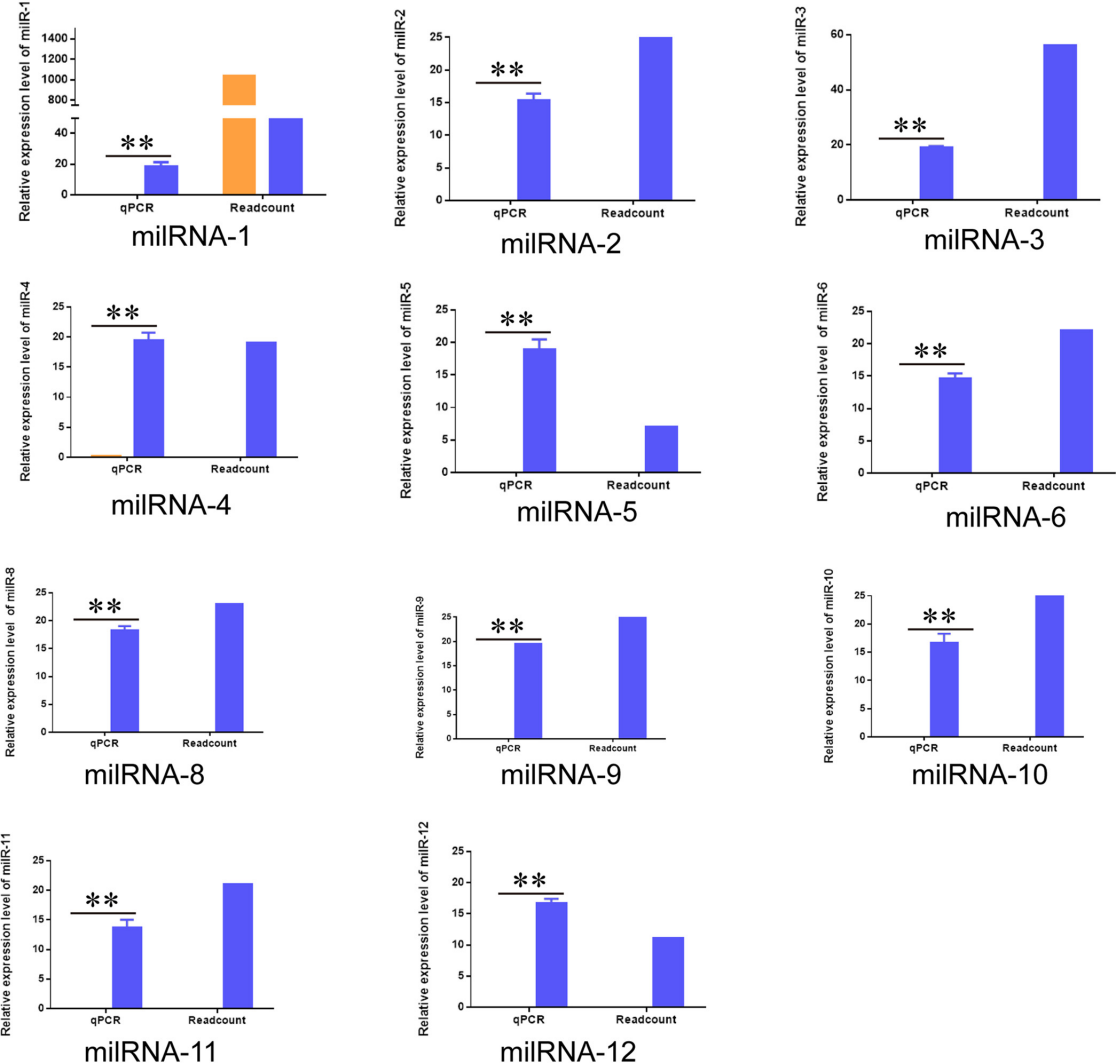
Transcriptional expression analysis of Nb-milRNAs. Normalized read numbers of Nb-milRNAs as transcripts per million reads (TPM) of ≥10 are shown for Nb (blue) or the control (orange) groups (i.e., “readcount” on right) in addition to expression of milRNAs that was evaluated with stem-loop RT-PCR (i.e., “qPCR” on right). All data represent the means of three replicates ± SD. **, *P < *0.01.

To identify milRNAs via high-throughput sequencing, total miRNAs at 48 h p.i. were extracted, and the expressions of predicted miRNAs were evaluated with stem-loop RT-PCR. The expression levels of the 11 milRNAs verified by stem-loop RT-PCR were consistent with their expression trends based on high-throughput sequencing and were only highly expressed in host cells infected with *N. bombycis*. Although Nb-mil1 was predicted to be expressed in infected and uninfected samples, it was also only expressed in host cells after infection with *N. bombycis* (Fig. 3).

### Functional assessment of *N. bombycis* Nb-milR8.

**FIG 4:**
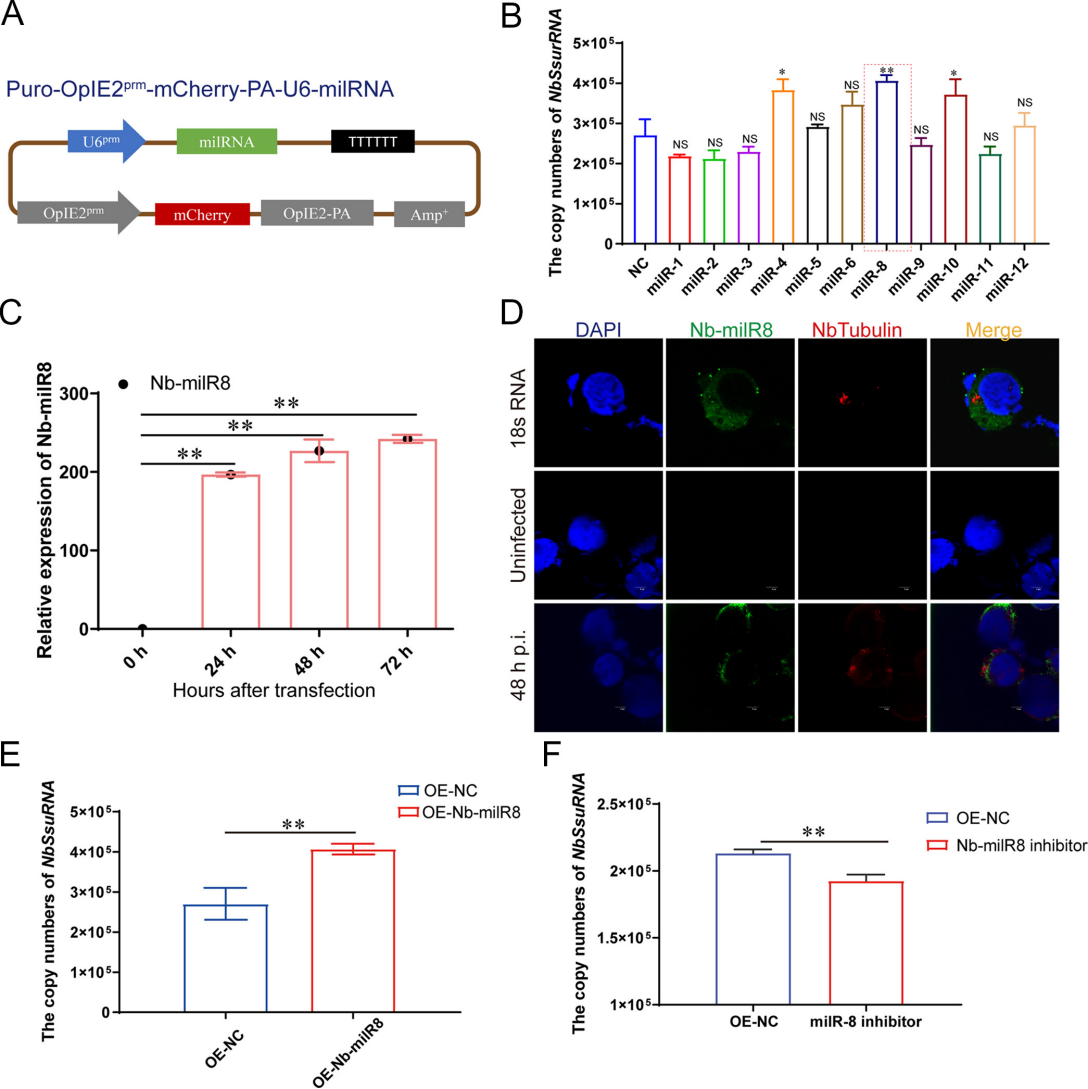
Functional assessment of Nb-milR8. (A) Schematic showing the construction of the Nb-milRNA overexpression vector. (B) Effects of milRNAs on *N. bombycis* proliferation, as determined by genome copy numbers. Statistically significant differences were determined using Student’s *t* test; **, *P < *0.01; *, *P < *0.05; NS, ≥0.05. (C) Transcriptional levels of Nb-milR8 in BmN-SWU1 cells that were challenged with *N. bombycis*. (D) Localization of Nb-milR8 based on fluorescence *in situ* hybridization (FISH). DAPI shows the *B. mori* and *N. bombycis* nuclei; red fluorescence represents the Nb-tubulin protein; green represents the fluorescence probe for Nb-milR8. (E) Genomic DNA copy enumeration during Nb-milR8 overexpression compared to the control transformant. OE-NC indicates overexpression of the native control plasmid Puro-OpIE2^prm^-mCherry-PA. (F) Genomic DNA copy enumeration with the Nb-milR8 inhibitor compared to that with the control transformant. OE-NC indicates overexpression of the native control plasmid Puro-OpIE2^prm^-mCherry-PA. Statistically significant differences were determined using Student’s *t* test; **, *P < *0.01. All data represent the means of three replicates.

To explore the function of milRNAs on *N. bombycis* proliferation, target bands containing 11 Nb-milRNAs were inserted into Puro-OpIE2^prm^-mCherry-PA vectors to obtain recombinant Puro-OpIE2^prm^-mCherry-PA-U6-milRNA plasmids that would enable Nb-milRNA expression detection with fluorescence microscopy (Fig. 4A). After transfection with miRNAs into the BmE-SWU1 cells, Nb-milR4, Nb-milR8, and Nb-milR10 promoted the proliferation of *N. bombycis*, while Nb-milR1, Nb-milR2, Nb-milR3, Nb-milR5, Nb-milR6, Nb-milR9, Nb-milR11, and Nb-milR12 did not exhibit significant differences upon *N. bombycis* proliferation. Among these, Nb-milR8 was most effective toward changes in pathogen infection (Fig. 4B).

To analyze Nb-milR8 expressional characteristics, stem-loop RT-PCR was used to analyze its expression profiles. Nb-milR8 expression levels increased after *N. bombycis* infection (Fig. 4C). A specific fluorescent *in situ* hybridization (FISH) probe was designed according to the nucleic acid sequence of Nb-milR8, allowing us to detect the Nb-milR8 expression characteristics after infection with *N. bombycis* within BmE-SWU1 cells. FISH analysis indicated that Nb-milR8 was expressed in the cytoplasm of *N. bombycis* and in host cells (Fig. 4D).

To further explore the effects of Nb-milR8 on *N. bombycis* proliferation, its effects on *N. bombycis* were evaluated after overexpression. In addition, we artificially designed an Nb-milR8 inhibitor to inhibit Nb-milR8 expression. After infection with *N. bombycis*, RT-PCR was used to analyze the genomic DNA copy numbers of *N. bombycis*. Overexpression of Nb-milR8 promoted the proliferation of *N. bombycis*, and Nb-milR8 inhibitor was effective at inhibiting *N. bombycis* proliferation (Fig. 4E and F). These results thus indicate that Nb-milR8 plays an important role in *N. bombycis* proliferation.

### Regulation of target gene expression by Nb-milR8.

**FIG 5:**
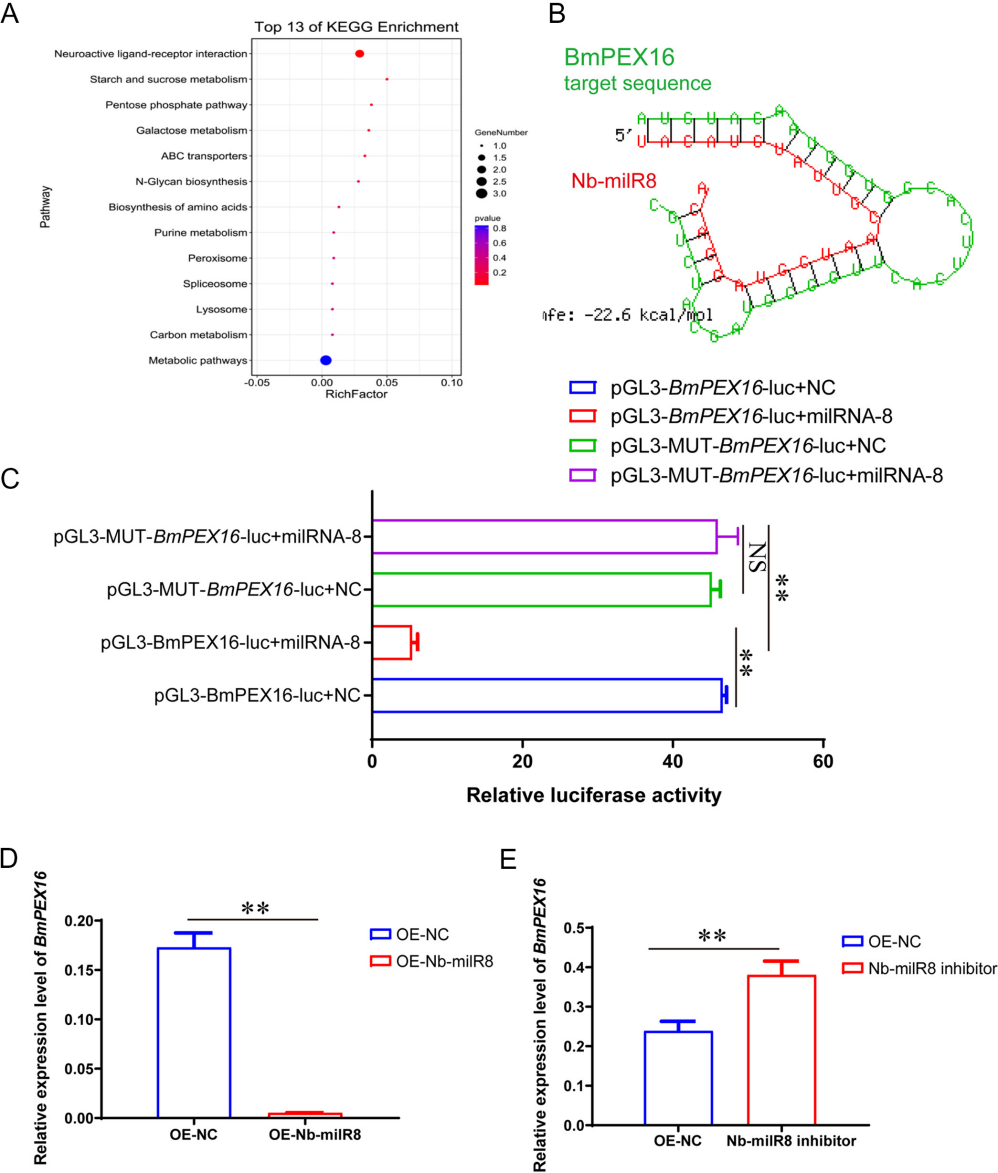
Regulation of Nb-milR8 target gene expression. (A) KEGG enrichment analysis for the Nb-milR8 target gene. (B) Target gene prediction for Nb-milR8 was performed using the RNA hybrid program and is shown in the schematic. (C) Dual-luciferase reporter assay for the Nb-milR8 binding site. OE-NC indicates overexpression of the native control plasmid Puro-OpIE2^prm^-mCherry-PA. pGL3-MUT-BmPEX16-luc indicates mutation of the *BmPEX16* gene bind site. (D) Relative expression of *BmPEX16* after overexpression with Nb-milR8. (E) Relative expression of *BmPEX16* after transfection with the Nb-milR8 inhibitor. OE-NC indicates overexpression of the native control plasmid Puro-OpIE2^prm^-mCherry-PA. All data represent the means of three replicates ± SD. **, *P < *0.01.

To identify the target gene of Nb-milR8 in Bombyx mori, the target gene prediction software program Miranda was used for preliminary predictions, with a threshold score set at ≥140. A total of 569 target genes were predicted for Nb-milR8 in Bombyx mori. Among these genes, 64 exhibited expression differences of over 2-fold when comparing the control and Nb groups (Fig. 2S). KEGG functional enrichment analysis indicated that eight target genes were enriched, and these were mainly associated with metabolism, neuroactive receptor ligand pathways, and peroxisome pathways (Fig. 5A). The expression levels of five of these eight genes were significantly upregulated, one gene did not exhibit significant expression differences, and two exhibited significantly downregulated expression after overexpression of Nb-milR8 (Fig. 3S). The most significant downregulated gene, *BmPEX16* (NCBI reference sequence: XM_012696074.3), was chosen as a primary candidate target gene.

The binding of Nb-milR8 and *BmPEX16* was explored using the Miranda program. The seed sequence of Nb-milR8 complemented and paired with the 3′ untranscribed region (UTR) region of *BmPEX16* (Fig. 5B). To further verify the binding effects of Nb-milR8 and BmPEX16, Nb-milR8 and pGL3.0-IE1-Luc-PEX16 (or a native control and pGL3.0-IE1-Luc-PEX16) were transfected into BmE-SWU1 cells, respectively. Then, 48 h posttransfection (p.t.), *BmPEX16* expression was clearly regulated by Nb-milR8, and firefly luciferase activity decreased. In addition, the activity of dual luciferase decreased, indicating that *BmPEX16* is indeed the target gene of Nb-milR8 and is regulated by Nb-milR8 (Fig. 5C). In contrast, no significant changes in luciferase activity were observed compared to the control after mutation of the *BmPEX16* binding site. To further explore the regulatory effects of Nb-milR8 on *BmPEX16*, the synthetic Nb-milR8 inhibitor and the Nb-milR8 vectors were overexpressed in BmN-SWU1 cells, respectively. In addition, the expression of *BmPEX16* was measured with RT-PCR. The Nb-milR8 inhibitor upregulated the expression level of the target gene after infection with *N. bombycis*, and overexpression of Nb-milR8 inhibited the expression level of the target gene (Fig. 5D and E). Thus, these results demonstrate that *BmPEX16* is the target gene of Nb-milR8 and is regulated by it.

### The target gene *BmPEX16* of Nb-milR8 is required for *N. bombycis* proliferation.

**FIG 6:**
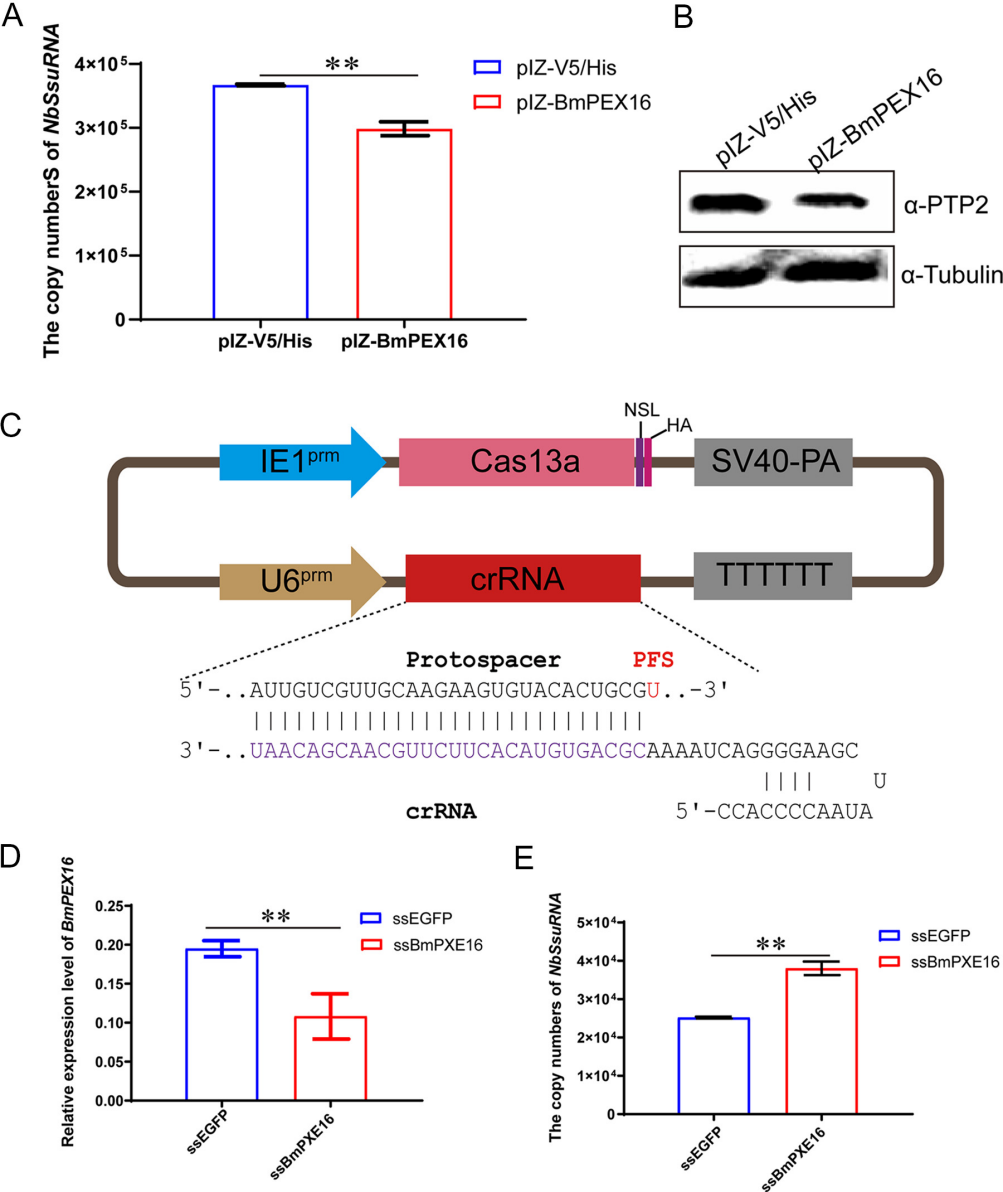
The target gene of Nb-milR8, *BmPEX16*, is required for *N. Bombycis* proliferation. (A) Analysis of *N. bombycis* genome copies after overexpression with *BmPEX16*. (B) NbPTP2 protein expression analysis after overexpression with *BmPEX16* based on Western blot analysis. (C) Schematic showing the construction of a Cas13a gene editing system in *B. mori*. (D) Knockout efficiency of *BmPEX16*. (E) Genomic DNA copies of *N. bombycis* after knockout of *BmPEX16*. All data represent the means of three replicates ± SD. **, *P < *0.01.

PEX16 is essential for peroxisome membrane biogenesis and may play a role in the early stages of peroxisome assembly (24, 25). To analyze peroxisome function during *N. bombycis* proliferation, a *BmPEX16* overexpression vector was constructed and transfected into BmE-SWU1 cells. DNA and proteins were extracted from the samples after infection of *N. bombycis* at 48 h p.i. *N. bombycis* DNA copy numbers and NbPTP2 protein levels of *N. bombycis* were detected by quantitative PCR (qPCR) and Western blotting, respectively. Genome copy numbers for *N. bombycis* were downregulated, while protein expression of NbPTP2 was also downregulated after overexpressing the target gene *BmPEX16*. These results indicated that the proliferation of *N. bombycis* was inhibited by *BmPEX16* expression (Fig. 6A and B).

To analyze the inhibitory effect of Nb-milR8 on *BmPEX16* expression, a *BmPEX16* knockout vector was constructed using CRISPR/Cas13 technology (Fig. 6C). After transfecting with PSL1180-IE1-Cas13a-SV40-U6-ssPEX16, the expression of *BmPEX16* was significantly reduced, as indicated by RT-PCR (Fig. 6D). To confirm the effects of the *BmPEX16* knockout on *N. bombycis* proliferation, PSL1180-IE1-Cas13a-SV40-U6-ssPEX16 was transfected into cells for 48 h. Then, 48 h p.i., copy numbers were detected by RT-PCR, revealing that *N. bombycis* genome copy numbers were upregulated after knockout of the target gene *BmPEX16* using the CRISPR/Cas13a system. Together, these results indicate that Nb-milR8 inhibited *BmPEX16* expression by targeting its binding site and promoting the proliferation of *N. bombycis*.

## DISCUSSION

Following the discovery of the first miRNA, *lin4*, in Caenorhabditis elegans in 1993, the development of biomolecular methods and bioinformatics has led to increasing numbers of miRNAs being identified in animals, plants, viruses, and even some single-celled organisms (12, 19, 26, 27). However, few milRNAs (microRNA-like RNAs) have been reported in fungi, and the mechanisms of their actions within cells are largely unknown. Microsporidia are evolutionarily unique pathogenic fungi (20). A functional RNA interference (RNAi) pathway was recently identified in the microsporidial fungus Nosema ceranae, indicating that miRNAs also exist in microsporidia (2, 28). An understanding of milRNA functions in the context of these organisms’ position within eukaryotes, in addition to the treatment of microsporidia infections, are areas of considerable interest. In this study, numerous sRNAs were identified in the schizonts of *N. bombycis* using high-throughput whole-genome sequencing, with additional characterization of 11 Nb-milRNA’s expression profiles (Fig. 3). Moreover, the mechanism underlying the promotion of *N. bombycis* proliferation by Nb-milR8 was identified as involving the regulation of the target host peroxisomal membrane protein *BmPXE16* gene.

The development of high-throughput sequencing technology has led to the identification of milRNAs in Fusarium oxysporum, Rhizoctonia solani, Cordyceps guangdongensis, and Volvariella volvacea, while also considerably promoting the discovery of milRNAs with low expression and those that exhibit tissue-specific expression (29–31). In this study, the schizont phase of microsporidian infection was chosen for characterization, and a total of 11 mature milRNAs were identified and mapped, in addition to 1,063 sRNA types. Thus, milRNAs only composed 0.24% of the total sRNA pool (Fig. 2B). Likewise, only six milRNAs were identified in the microsporidian parasite Nosema ceranae, with only three being further validated, which is relatively minimal compared to other fungi (27, 32). BLAST searches of the milRNAs identified here also did not yield highly homologous milRNAs outside *N. bombycis*, indicating that the milRNAs arose independently from the evolution of plant, animal, and fungal miRNAs. Taken together, 11 milRNAs were validated in this study, thereby increasing the number of Nb-milRNAs known in microsporidian parasites and providing baseline data to support future research into microsporidian Nb-milRNA.

Functional RNAi pathways, *Dicer*, and *Argonaute*-gene based pathways have been documented as infection strategies promoting the selectivity of microsporidian parasitism (2). One of the most important discoveries of this study is that of diverse pathways for small RNA biogenesis within *N. bombycis*, including those for rRNA, tRNA, snRNA, snoRNA, milRNA, Dicer-independent siRNA (disiRNA), and small interfering RNA (siRNA) pathways (Fig. 2B). Our investigation of 11 Nb-milRNAs indicates that they are involved in regulating fungal virulence, suggesting that the biosynthesis pathway of milRNAs in *N. bombycis* may be similar to that of other fungi (Fig. 4). Pathogenic milRNAs are generally considered necessary components for host-pathogen interactions, with milRNAs generally thought to be helpful for promoting pathogen proliferation and replication (8, 17). For example, Vm-milRNAs adaptively regulate the virulence genes encoding sucrose nonfermenting 1 (VmSNF1), 4,5-DOPA dioxygenase extradiol (VmDODA), and a hypothetical protein (VmHy1) that enhances the virulence of *V. mali* in apple trees. Further, Pst-milRNA1 plays a role in virulence by inhibiting wheat pathogenicity-related genes that impair wheat resistance to Puccinia striiformis *f.* sp. *tritici*. Likewise, Vd-milR1 regulates the virulence of Verticillium dahlia through epigenetic inhibition of target gene expression (16, 19, 33). Here, we identified 11 Nb-milRNAs associated with pathogenicity of *N. bombycis* and confirmed the mechanism of Nb-milR8 promotion of *N. bombycis* proliferation via negative regulation of the peroxisome metabolic pathway through *BmPXE16* gene expression (Fig. 4 and 5). The results provide insight into a unique strategy to successfully develop microsporidian parasitism of hosts wherein pathogenic fungi must disable host defenses and hijack host metabolisms.

**FIG 7:**
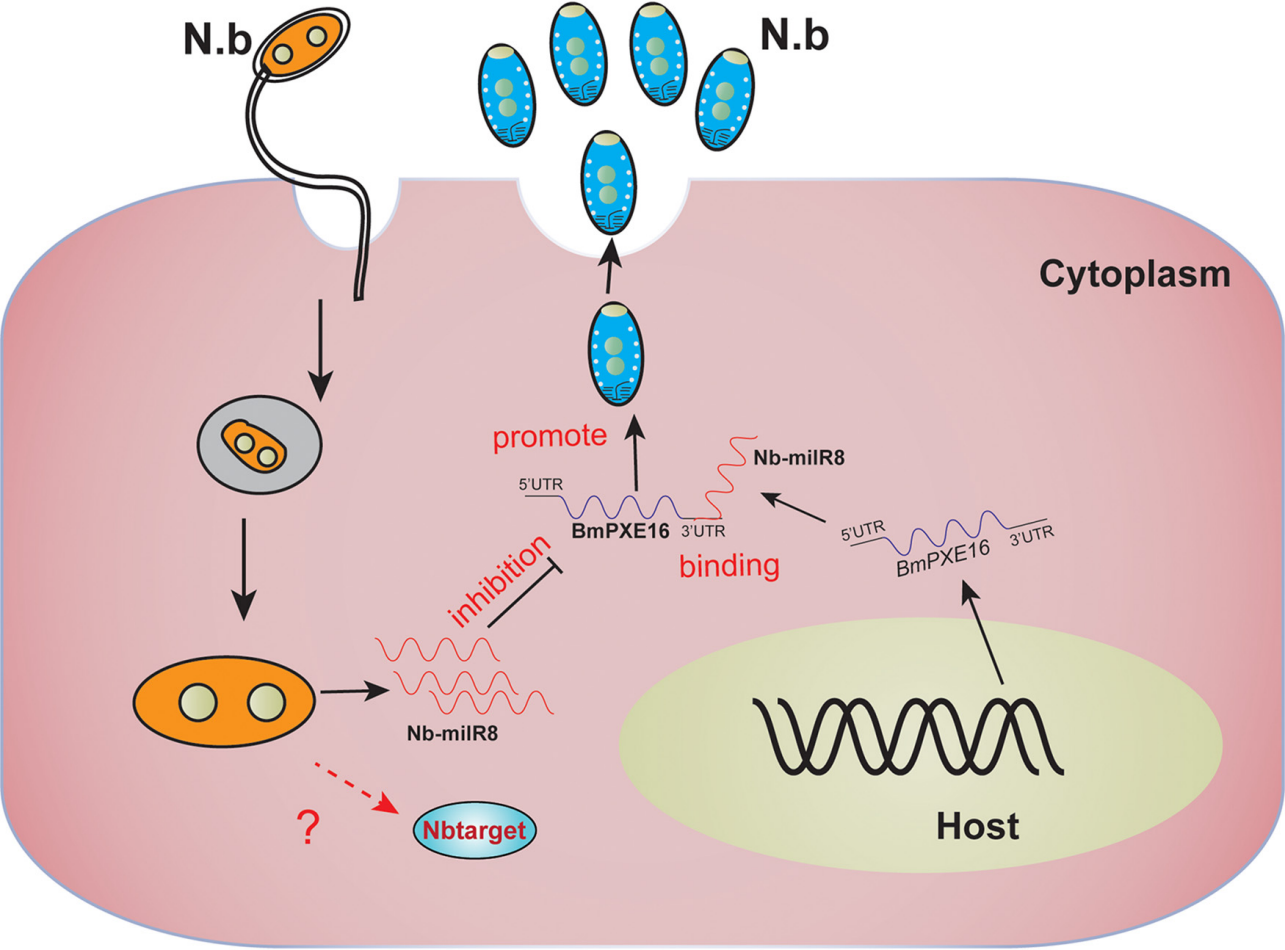
Model for *N. bombycis* deployment of Nb-milR8 to promote infection by modulating *BmPEX16* gene expression.

milRNAs play important roles during fungal infection by regulating the translation or mRNA stability of target genes (14, 30). Nevertheless, the lack of effective methods to identify the target genes of milRNA has led to limited functional exploration of milRNAs in fungi. In this study, a total of 569 target genes of *B. mori* and 110 target genes of *N. bombycis* were predicted for Nb-milR8, and their functional annotation revealed diverse activities in energy metabolism and peroxisome pathways (Fig. 5). To identify the interaction mechanism between the microsporidian parasite and its host, eight target genes of *B. mori* were identified by differential expression characteristics and functional enrichment (Fig. S2). *PEX16* genes are important regulators of peroxisome biosynthesis and are involved in peroxisome formation and the energy metabolism of the yeasts *Yarrowia* and Saccharomyces cerevisiae (24, 34–36). Here, we also demonstrated that Nb-milR8 negatively regulated the *BmPEX16* gene promoter to encourage *N. bombycis* proliferation during infection. These results led to the hypothesis that upregulated expression of Nb-milR8 after infection with *N. bombycis* leads to downregulated expression of *BmPEX16* by targeting the *BmPEX16* 3′ UTR, thereby ultimately inhibiting host *BmPEX16* expression and promoting the proliferation of *N. bombycis* (Fig. 7). These studies provide important target milRNAs for genetically engineering microsporidia. Infection-resistance materials could be further developed for transgenetic overexpression of milRNAs to inhibit *N. bombycis* proliferation or via the interference of milRNA that could then promote *N. bombycis* proliferation. In addition, resistance mechanisms could be targeted by focusing on the Nb-milRNA encoding gene to improve silkworm resistance to *N. bombycis* infection.

### Conclusions.

This study demonstrated that *N. bombycis* regulated its host peroxisome pathway *BmPEX16* gene via the Nb-milR8 RNA interacting with its promoter, thus enabling *N. bombycis* proliferation during infection. These results illustrate a regulatory mechanism of Nb-milR8 toward host metabolism that occurs during host-pathogen interactions. Consequently, these results deepen our understanding of mechanistic interactions between hosts and pathogens while providing new insights into the possible prevention of *N. bombycis* fungal infections.

## MATERIALS AND METHODS

### *B. mori* cell and *N. bombycis*.

BmE-SWU1 cell lines were cultured at 27°C in Grace medium (United States Biological, USA) (37). Cell culture medium comprised 90% Grace free medium and 10% fetal bovine serum (FBS) (Gibco, USA). The *N. bombycis* strain CQ1 (CVCC 102059) was obtained from the China Veterinary Culture Collection Center.

### RNA extraction and sequence data analysis.

Total RNAs were extracted from the control (0 h p.i.) and the *N. bombycis* infection group (48 h p.i.) using an RNA isolation kit according to the manufacturer’s instructions (Omega, USA). After sample quality verification, a small RNA sample kit was used to construct a sequencing library (Yeasen, China). The unique structures of the 3′ and 5′ ends of small RNAs (i.e., 5′ ends with complete phosphate groups and 3′ ends with hydroxyl groups) allowed the use of total RNA as the starting samples, followed by direct splicing of small RNA ends and production of cDNA via reverse transcription. After PCR amplification and PAGE gel electrophoresis, a cDNA library was obtained. The different libraries were pooled and sequenced on the Illumina HiSeq TM2000/MiSeq sequencing platforms based on the effective concentrations and the requirements for target data quantities. Raw reads were first processed to obtain high-quality clean reads. The raw sequence reads will be available through the NCBI database under the accession number PRJNA760284. sRNAs of a certain length range were screened for subsequent analyses from the clean read data sets for each sample. The small RNA tags were then mapped to reference sequences using Bowtie after specifying a threshold for sequence mismatches in order to analyze their expression profiles against the reference (38).

### Identification of milRNAs.

Few fungal miRNA gene data sets are present in the miRbase database, and thus characteristics of miRNA precursor hairpin structures were used to predict novel miRNAs. Specifically, the miREvo and mirdeep2 software programs were both used to predict novel miRNAs by exploring secondary structures, Dicer cleavage sites, and minimum free energy values of small RNA tags that were not annotated as described previously (22, 23). Further, custom scripts were used to obtain miRNA counts for different sequence length RNAs, in addition to base biases at each position for all identified miRNAs. The target gene prediction software program Miranda was used for preliminary predictions of Nb-milR8 target genes in *N. bombycis*, specifying a Miranda threshold score of ≥140.

### miRNA extraction and expression analysis.

Total miRNAs at 48 h p.i. were extracted using a Molpure cell/tissue miRNA kit (Yeasen, China) according to the manufacturer’s protocols. The RNA was reverse transcribed using a specific stem-loop primer, and miRNA cDNA was used for hydrolysis probe-based RT-PCR. Primer sequences are provided in Table S1. The relative changes in expression values among groups were calculated using the 2^–△^*^CT^* method.

### Scanning electronic microscopy (SEM).

BmE-SWU1 cells were infected with *N. bombycis* for 0, 3, 9, 48, 72, and 84 h, and samples were fixed for SEM analysis. Specifically, the samples were sectioned to about 10 nm with a slicer microtome instrument and then stained with acetic and citric acid before imaging with a Hitachi-SU3500 SEM instrument (SU3500, Hitachi, Japan). Images were viewed at an accelerating voltage of 100 kV using a low beam current.

### Construction of milRNA expression plasmids.

To construct milRNAs, specific primers for downstream regions of milRNA were designed according to milRNA sequences. milRNAs were PCR amplified using U6 as the template. The amplified target bands of U6-milRNA and Puro-OpIE2^prm^-mCherry-PA were then digested using the *Asc* I restriction endonuclease and then ligated with linkers for 24 h. The Nb-milR8 inhibitor exhibits a complementary pairing sequence for Nb-milR8. After synthesis, the construct was similarly ligated to the Puro-OpIE2^prm^-mCherry-PA vector to enable competitive binding of Nb-milR8 with the target gene. The Puro-OpIE2^prm^-mCherry-PA-U6-milRNA plasmids were then subjected to sequencing.

### Construction of Cas13a gene editing vectors.

To knockout the *BmPEX16* gene of *B. mori*, wild-type LwCas13a and pC014 (pC014-LwCas13a-msfGFP, Addgene plasmid no. 91902) plasmids were obtained from Addgene (39). The LwCas13a fragments were cloned into pSL1180-IE1^prm^-SV40 vectors by digesting at the *Xho* I and *Not* I restriction sites, ultimately yielding the pSL1180-IE1^prm^-LwCas13a-SV40 product. Candidate ssRNA target sequences were designed using the CRISPR design software program (http://bioinfolab.miamioh.edu/CRISPR-RT/interface/C2c2.php), and U6-ssRNA expression cassettes were linked into the pSL1180-IE1^prm^-LwCas13a-SV40.

### Dual luciferase reporter assays.

The dual luciferase expression plasmids pGL3-IE1-Fluc-PEX16 (i.e., the 3′ UTR of the target gene fused at the 3′ end of the firefly luciferase gene), pGL3-IE1-Rluc (with Rluc as an internal reference gene), and milRNA relative vectors were cotransfected into BmN-SWU1 cells. Cells were then collected at 48 h posttransfection (h p.t.), and luciferase activities were measured using a Dual-Glo luciferase assay kit (Promega, USA). Relative luciferase activity (Fluc/Rluc) was normalized to values obtained with pGL3-Basic as the control plasmid.

### Immunofluorescence.

After *N. bombycis* infection of BmN-SWU1 cells at 24, 48, 72, and 96 h, samples were seeded on cover glasses for immunofluorescence assays (Thermo Scientific, USA) in 24-well plates (Corning, NY, USA) (40). In addition, *N. bombycis* at 24, 48, 72, and 96 h p.i., was stained with anti-Nb tubulin antibody and an Alexa 488 conjugated goat anti-rabbit secondary antibody. Similarly, localization of Nb-milR8 was evaluated after incubation with Nb-milR8 probes followed by fluorescence *in situ* hybridization (FISH) analysis. *N. bombycis* nuclei were then stained with DAPI for 1 h. All images were visualized with an Olympus inverted fluorescence microscope at the corresponding excitation wavelengths.

### Western blotting.

Overexpression of pIZ-BmPEX16 and pIZ-V5/His vectors was induced in BmN-SWU1 cells. After 48 h p.t., samples were lysed using immunoprecipitation (IP) buffer to enable Western blot analysis (Beyotime, China). Total sample protein extracts were separated by SDS-PAGE and then blot-transferred to polyvinylidene fluoride membranes. The membranes were then incubated with rabbit α-tubulin (1:2,000) and rabbit α-PTP2 (1:2,000), followed by further incubation with horseradish peroxidase (HRP)-labeled goat anti-mouse or anti-rabbit IgG (1:5,000; Beyotime, China). Bands were visualized using the Clarity Western ECL substrate (Bio-Rad, USA).

### Quantitative PCR (qPCR).

The qPCRs were conducted in a CFX96 real-time system using the SYBR select master mix reagent (Bio-Rad, Hercules, CA, USA) to determine *N. bombycis* genome copy numbers. Specifically, *N. bombycis* small subunit rRNA (*SSUrRNA*) gene-specific primers were used to amplify rRNA genes and estimate genome replication to ultimately evaluate genome copy numbers after 48 h of infection (41). A standard curve described by *y* = −3.4305× + 36.882 (*R*^2^ = 0.9989) was used to evaluate the expression levels. The sw25113 gene was used as an internal reference to determine the relative gene expression from qPCRs data. qPCRs were conducted at 95°C for 30 s, followed by 40 cycles of 95°C for 5 s and 60°C for 30 s.

### Statistical analyses.

Results are expressed as the means ± standard deviations (SD). A value of *P < *0.01 (**) based on two-tailed tests was considered statistically significant for all analyses. All statistical analyses were conducted using the GraphPad Prism 8 software program.
