# Supplementary material for: Nosema bombycis microRNA-like RNA 8 (Nb-milR8) Increases Fungal Pathogenicity by Modulating BmPEX16 Gene Expression in Its Host, Bombyx mori
Source: Microbiol Spectr. 2021 Oct 27;9(2):e01048-21. doi: 10.1128/Spectrum.01048-21 (PMC8549759; doi:10.1128/Spectrum.01048-21)
Supplement: SUPPLEMENTAL FILE 1 — Supplemental material. Download SPECTRUM01048-21_Supp_1_seq11.pdf, PDF file, 0.4 MB [file spectrum01048-21_supp_1_seq11.pdf]

Supplementary material

Nosema bombycis microRNA-like RNA 8 (Nb-milR8) increases fungal pathogenicity by modulating *BmPEX16* gene expression in its host, *Bombyx mori*

Zhanqi Dong[1,2†], Ning Zheng[1†], Congwu Hu[1], Boyuan Deng[1], Wenxuan Fang[1], Qin Wu[1], Peng Chen[1,2], Xuhua Huang[3], Na Gao[1†], Cheng Lu[1,2‡], Minhui Pan[1,2‡]

(1. State Key Laboratory of Silkworm Genome Biology, Southwest University, Chongqing 400716, China;

2. Key Laboratory of Sericultural Biology and Genetic Breeding, Ministry of Agriculture and Rural Affairs, Southwest University, Chongqing 400716, China;

3. The General Extension Station of Sericulture Technology of Guangxi Zhuang Autonomous Region，Nanning 530007，China.)

[†]These authors contributed equally to this work.

[‡]Address correspondence to Cheng Lu, Tel: +86-023-68250346, Fax: +86-023-68251128, E-mail: lucheng@swu.edu.cn, and Min-Hui Pan, Tel: +86-023-68250076, Fax: +86-23-68251128, E-mail: pmh047@ 126.com.

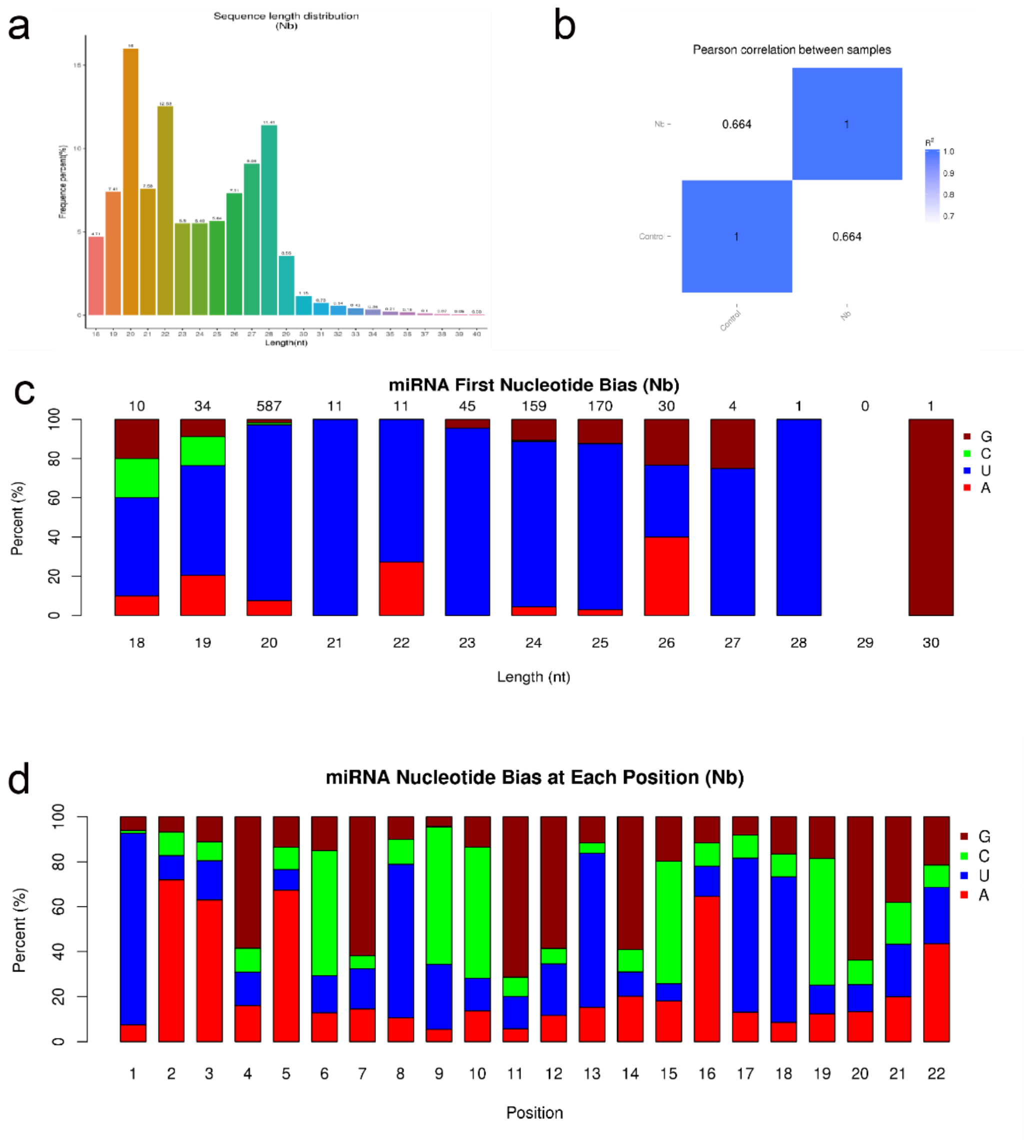

Fig. S1. Characterization of milRNAs in the Nb group treatment.

(A) The length distribution of sRNAs based on small RNA sequences in the Nb group sequencing libraries. (B) Heatmap of milRNA expression among samples. (C) First nucleotide bias of milRNAs identified in *N. bombycis*. (D) Nucleotide bias at each position within milRNA candidates in *N. bombycis*.

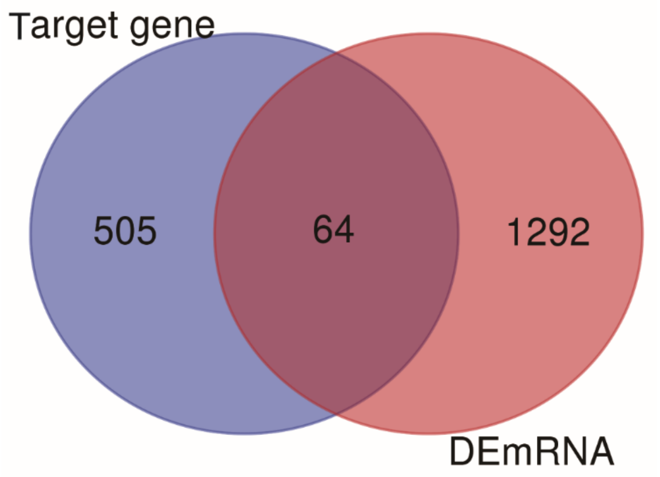

Fig. S2. Venn Diagram depicting the Nb-milR8 targeted genes and significantly downregulated gene after *N. bombycis* infection. Target gene indicate 569 target genes were predicted for Nb-milR8 in *Bombyx mori*. DEmRNA indicate a difference comparative threshold of ≥ 2 between the control group and the N.b group.

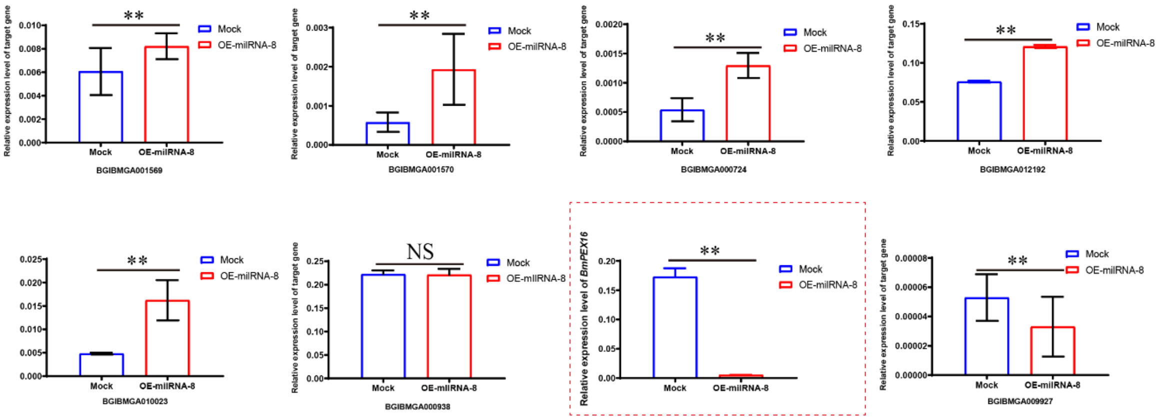

Fig. S3. Relative expression of target genes after overexpression of Nb-milR8. All data represent means of three replicates ± SD. **: $p < 0.01$.

Supplementary Table 1. Primers used in this study.

Name	序列
BmActin/F	CATGAAGATCCTCACCGAGCG
BmActin/F	CGTAGCACAGCTTCTCCTTGATA
NbssuRNA/F	GTCCCTGTTCTTTGTAC

NbssuRNA/R	ATCCTGCTAATGGTTCT
U6/F	CTCGCTTCGGCAGCACA
U6/R	AACGCTTCACGAATTTGCGT
milRNA-1/F	CTCAACTGGTGTCGTGGAGTCGGCAATTCAGTTGA GCGAATGCA
milRNA-1/R	ACACTCCAGCTGGGTAAGACGTCCGG
milRNA-2/F	CTCAACTGGTGTCGTGGAGTCGGCAATTCAGTTGA GGGCTCGTC
milRNA-2/R	ACACTCCAGCTGGGTATTATTGTAGTTAGAA
milRNA-3/F	CTCAACTGGTGTCGTGGAGTCGGCAATTCAGTTGA GTCAACCGC
milRNA-3/R	ACACTCCAGCTGGGTAAAGATTTTCATAGA
milRNA-4/F	CTCAACTGGTGTCGTGGAGTCGGCAATTCAGTTGA GTCGTCCAG
milRNA-4/R	ACACTCCAGCTGGGTTTAGCGTCGTAAAGTG
milRNA-5/F	CTCAACTGGTGTCGTGGAGTCGGCAATTCAGTTGA GTGTGAACG
milRNA-5/R	ACACTCCAGCTGGGTCGGCGTTGTGGTTTA
milRNA-6/F	CTCAACTGGTGTCGTGGAGTCGGCAATTCAGTTGA GAAGCTTCA
milRNA-6/R	ACACTCCAGCTGGGTATTTAGATCAAAGGTT
milRNA-8/F	CTCAACTGGTGTCGTGGAGTCGGCAATTCAGTTGA GTGTCCTAC
milRNA-8/R	ACACTCCAGCTGGGTACATGTATTGCAATC
milRNA-9/F	CTCAACTGGTGTCGTGGAGTCGGCAATTCAGTTGA GAATCTACA
milRNA-9/R	ACACTCCAGCTGGGTTCCGAAATCGTCTGCT
milRNA-10/F	CTCAACTGGTGTCGTGGAGTCGGCAATTCAGTTGA GATGTCAGG
milRNA-10/R	ACACTCCAGCTGGGTGACATGCTGTTAAA
milRNA-11/F	CTCAACTGGTGTCGTGGAGTCGGCAATTCAGTTGA GGGTTTACG
milRNA-11/R	ACACTCCAGCTGGGTTTCCGATATTTTGGG
milRNA-12/F	CTCAACTGGTGTCGTGGAGTCGGCAATTCAGTTGA

	GTTGCCAGA
milRNA-12/R	ACACTCCAGCTGGGTCTTTGCTGTAATGTT
OE-BmPX16/F	CTAGCGTCTACGATGCGGTTCACTTCACGGTGGTA
OE-BmPX16/R	ACATGTAACCGG
	TTACATGTTACCACCGTGAAGTGAACCGCATCGTA
	GACG
	ACTTACGCTGAGTACTTCGAAATGT
	CGTGAAGTGAACCGCATCGTAGACG
BGIBMGA002029/F	AAGTATACTGAAGGCCGAGATG
BGIBMGA002029/R	TCCATCAACACGTCTCATAACA
BGIBMGA009927/F	CTCTGCGAAGAGTTCAACAATC
BGIBMGA009927/R	GAACGAACTCAGGACAGGAA
BGIBMGA010023/F	CCAACTTCCATATCCTGTCTGT
BGIBMGA010023/R	TCATTTCGTATGTCAGAACCGA
BGIBMGA001569/F	AATGGAGCAAGAGGAAGAAGAA
BGIBMGA001569/R	TCATCCAAAGGCCATGTATCTT
BGIBMGA001570/F	CGTTAGAGGTGAGGATGTCTAC
BGIBMGA001570/R	TTTCTTGTCCTGTCTCGAGTAC
BGIBMGA000724/F	GTTACGGAAATTGTTTGGAGCT
BGIBMGA000724/R	GTTAACCAATCTTTTGCCACCT
BGIBMGA000938/F	AGAGGCGAGTTGTGTTTATACA
BGIBMGA000938/R	CTTCTGCAGGAAGTACTTCTGA
BGIBMGA012192/F	ATTCGTGAGACAAAAGAAACCG
BGIBMGA012192/R	ACCTTTAACATTTCCTGGTCCT
Cas13a/F	ACCAAACGACTAGGGATCTACTCGAGATGTATCCG
	TATGACGTCC
Cas13a/R	TGGTATGGCTGATTATGATCGCGGCCGCTCACTTCT
	TCTTCTTAG
ssBmPEX16/F	AAACCGCAGTGTACACTTCTTGCAACGACAAT
ssBmPEX16/R	AAAA ATTGTCGTTGCAAGAAGTGTACACTGC

* (The restriction enzyme sites are marked in lower case.)

Table S2. Predicted sequence of *N. bombycis*-encoded milRNAs

milRNA	Sequence (5’-3’)	Length	Reads

		(nt)	
Nb-milR1	UAAGACGUCCGGUGCAUUCG	20	604
Nb-milR2	UAUUAUUGUAGUUAGAAGACGAGCC	25	77
Nb-milR3	UAAAGAUUUUCAUAGAGCGGUUGA	24	56
Nb-milR4	UUUAGCGUCGUAAAGUGCUGGACGA	26	19
Nb-milR5	UCGGCGUUGUGGUUUACGUUCACA	24	7
Nb-milR6	UAUUUAGAUCAAAGGUUUGAAGCUU	25	22
Nb-milR8	UACAUGUAUUGCAAUCGUAGGACA	24	23
Nb-milR9	UUCCGAAAUCGUCUGCUUGUAGAUU	25	34
Nb-milR10	UGACAUGCUGUUAAACCUGACAUC	24	41
Nb-milR11	UUUCCGAUAUUUUGGGCGUAAACC	24	21
Nb-milR12	UCUUUGCUGUAAUGUUUCUGGCAA	24	11

Table S3. Library preparation quality statistics.

Sample	Raw reads	Error rate	Q20	Q30	GC content	Clean reads
Nb	126,573,70	0.01%	99.35%	97.71%	50.82%	12,081,520 (95.45%)
Control	19,108,751	0.01%	99.32%	97.48%	49.03%	18,766,136 (98.21%)
